# A role of STING signaling in obesity-induced lung inflammation

**DOI:** 10.1038/s41366-023-01272-x

**Published:** 2023-02-13

**Authors:** Yong Qi, Zhuhua Wu, Dan Chen, Li Zhu, Yunlei Yang

**Affiliations:** 1 Department of Pulmonary and Critical Care Medicine, Zhengzhou University People’s Hospital, Henan Provincial People’s Hospital, Zhengzhou, Henan 450003 China; 2 Department of Medicine Division of Endocrinology, Albert Einstein College of Medicine, Bronx, NY 10461 USA; 3 Department of Neuroscience, Albert Einstein College of Medicine, Bronx, NY 10461 USA; 4 Einstein-Mount Sinai Diabetes Research Center, Albert Einstein College of Medicine, Bronx, NY 10461 USA; 5 The Fleischer Institute for Diabetes and Metabolism, Albert Einstein College of Medicine, Bronx, NY 10461 USA

**Keywords:** Endocrinology, Cell biology

## Abstract

**Background:**

It is established that pulmonary disorders are comorbid with metabolic disorders such as obesity. Previous studies show that the stimulator of interferon genes (STING) signaling plays crucial roles in obesity-induced chronic inflammation via TANK-binding kinase 1 (TBK1) pathways. However, it remains unknown whether and how the STING signaling is implicated in the inflammatory processes in the lung in obesity.

**Methods:**

Human lung tissues were obtained from obese patients (*n* = 3) and controls (*n* = 3). Mice were fed with the high-fat diet or regular control diet to establish the diet-induced obese (DIO) and lean mice, and were treated with C-176 (a specific STING inhibitor) or vehicle respectively. The lung macrophages were exposed to palmitic acid (PA) in vitro. The levels of STING singaling and metabolic inflammation factors were detected and anlyzed.

**Results:**

We find that STING^+^/CD68^+^ macrophages are increased in lung tissues in patients with obesity. Our data also show that the expressions of STING and the levels of proinflammatory cytokines are increased both in lung tissues and bronchoalveolar lavage fluid (BALF) in obesity compared to controls, and inhibition of the STING blunted the obesity-induced lung inflammation. Mechanistically, our data demonstrate that the STING signaling pathway is involved in the PA-induced inflammation through the STING-TBK1-IRF3 (interferon regulatory factor 3)/NF-κB (nuclear factor kappa B) pathways in the lung macrophages.

**Conclusions:**

Our results collectively suggest that the STING signaling contributes to obesity-associated inflammation by stimulating proinflammatory processes in lung macrophages, one that may serve as a therapeutic target in ameliorating obesity-related lung dysfunctions.

## Introduction

The prevalence of obesity has risen to pandemic dimensions over the past 50 years [1, 2]. Obesity causes many comorbidities including respiratory diseases, such as pneumonia, bronchial asthma, pulmonary hypertension, ARDS (acute respiratory distress syndrome) and obstructive sleep apnea syndrome [3–6]. There is extensive evidence to show that lung homeostasis and inflammatory immune responses are profoundly disturbed in obesity [7–9]. Obesity is a complex metabolic condition that affects many physiological processes, including immune functions [10, 11]. It has been suggested that obesity is associated with low-grade chronic inflammatory processes [12, 13]. Previous studies demonstrate that lung inflammatory cell populations are altered in obesity with increased expressions of adhesion receptors, which might contribute to the susceptibility to lung disorders in obesity [9, 14]. However, the involved molecular and cellular mechanisms remain incompletely understood.

The transmembrane protein 173 (TMEM173) or the stimulator of interferon genes (STING), a ubiquitously expressed protein on the membrane of endoplasmic reticulum (ER), governs type I interferons (IFNs) by activating interferon regulatory factor 3 (IRF3) pathways and evokes a prominent pro-inflammatory cytokine response (e.g., tumor necrosis factor α [TNF-α] and interleukin 6 [IL-6]) through activating nuclear factor kappa B (NF-κB) [15, 16]. STING is involved in the inflammatory responses of lung diseases such as lung injury, pulmonary infection and pulmonary fibrosis [17–20]. STING activation by leaked mitochondrial DNA (mt-DNA) in stimulated macrophages by lipopolysaccharide (LPS) could further promote proptosis of macrophages, which subsequently exacerbated lung inflammatory injury [20]. Meanwhile, recent studies show that the STING pathway is activated in adipose tissues probably by the released mtDNA in obesity, leading to an increase in chronic sterile inflammatory response [21]. To our best knowledge, however, it remains unclear whether and how the STING pathway partakes in the inflammatory process in obesity-related lung disorders.

In the current study, we set off to define the upregulation of the STING expression in the lung macrophages in patients with obesity. We also provided the evidence to indicate the involvement of macrophage STING signaling pathway in obesity-related lung inflammation.

## Materials and methods

### Human lung tissue collections

Human lung tissue samples were obtained from patients that needed lung resectional surgery for diagnostic and/or therapeutic purposes due to pulmonary solitary nodule in Zhengzhou University People’s Hospital. Exclusion criteria were as follows: underlying lung diseases, infectious lung diseases; bronchial asthma and bronchodilation; chronic obstructive pulmonary disease; sleep apnea hypoventilation syndrome; interstitial lung disease; acute respiratory distress syndrome; pulmonary heart disease and respiratory failure; diabetes. According to the body mass index (BMI), the enrolled patients were divided into obesity (BMI ≥ 30.0 kg/m^2^) and normal weight group (≥18.5 kg/m^2^ to <25 kg/m^2^) (Table S1). There were no patients receiving chemotherapy or radiotherapy before the surgery or suffered from any other known chronic inflammatory condition. Specimens were dissected with a minimum distance to the tumor of 5 cm (avoiding areas involving tumors). The study was approved by Medical Ethics Committee of Henan Provincial People’s Hospital (Ethical Review 2017(45)).

### Human lung tissue immunofluorescence staining

Deparaffinized and dehydrated human lung sections were subjected to antigen retrieval with sodium citrate antigen retrieval solution (Bioss, C02-02002) and incubated with 10% fetal bovine serum (Bioss, C7074) for 1 h at room temperature for blocking the non-specific antigen. Then, the lung sections were incubated with CD68 (Santa Cruz, sc-20060, 1:100) and STING (Abcam, ab181125, 1:100) at 4 °C overnight. The lung sections were washed with 0.1% PBS-Triton X-100 (Bioss, C03-03001) and incubated with goat anti-mouse IgG-Alexa Fluor® 647 (Abcam, ab150115, 1:500) and goat anti-rabbit IgG-Alexa Fluor® 488(Abcam, ab150077, 1:500) was subsequently conducted on sections at room temperature in dark for 1 h, followed with DAPI staining. The slides were again mounted and photographed under microscope (BX53, OLYMPUS, Japan).

### Animal studies

Six-week-old male C57BL/6 J mice were purchased from Beijing Vital River Laboratory Animal Technology Co. Ltd. Mice were kept in a temperature-controlled room (23–24 °C) under standard 12-hour light/dark conditions. According to previous literature [22], mice were randomly divided into DIO (diet-induced obesity) group with high-fat diet (D12492, Research Diets Inc., 60% kcal fat) or lean group with regular chow diet (Beijing Keao Xieli Feed Co., Ltd, 12% kcal fat) for a total of 12 weeks. For C-176 treatment, after 8 weeks feeding with high-fat diet or regular chow diet, the mice were randomly divided into three groups including DIO/C-176, DIO/vehicle, and lean/vehicle. C-176 (MedChemExpress, HY-112906) was reconstituted in DMSO at 17.9 mg/mL and diluted in 40% PEG300 (MedChemExpress, HY-Y0873) and 5% Tween-80 (MedChemExpress, HY-Y1891). Mice received an intraperitoneal injection of 1 μmol C-176 or vehicle once every other day for an additional 9 weeks with high-fat diet or regular chow diet feeding, and then sacrificed for sampling. All procedures involving mice were approved by Animal Ethics Committee of Henan Provincial Institute for Food and Drug Control and the protocol was strictly aligned to the ethical guidelines of the experimental animals of Henan Provincial Institute for Food and Drug Control.

### Lung histology

Lung tissues were fixed in 4% paraformaldehyde for 24 h. Then lung tissues were embedded in paraffin and cut into 5-μm thick sections.The lung sections were further stained with hematoxylin (H8070, Solarbio) and eosin (A600190, Sangon Biotech) using previous protocols. Finally, sections were observed and photographed under the microscope (BX53, OLYMPUS, Japan).

### Cell culture and palmitic acid treatment

The RAW264.7 macrophage cell lines were purchased and cultured in Dulbecco’s modified Eagle’s medium (DMEM) (Gibco) supplemented with 10% fetal bovine serum (Sigma). The MH-S cell lines were purchased and cultured in RPMI 1640 medium (Gibco) supplemented with 10% fetal bovine serum (Sigma). All cultures contained 100 U/ml of penicillin and 100 μg/ml streptomycin, and were maintained in a 37 °C incubator with 5% CO_2_. Afterwards, cells were cultured for palmitic acid (PA, Sigma) treatment or transfections. PA was added to the culture medium by combining PA with bovine serum albumin (BSA, Sigma). Briefly, sodium salt PA was dissolved in ethanol at 65 °C for 15 min and then this was combined with fatty acid free-BSA at final concentrations of 0.6 mM. This stock solution was filter-sterilized and stored at −20 °C. The control condition included a solution of ethanol mixed with BSA at the same concentration as the PA solution.

### shRNA-induced STING gene silencing

For RNA silencing, the shRNA plasmid targeting murine STING and a negative control shRNA were designed and synthesized by Enscript Biotech Corporation. The MH-S cells and RAW264.7 macrophages were transfected with shRNA using the Lipofectamine 3000 Transfection Reagent (Invitrogen, USA) according to the manufacturer’s instructions. The sequences of STING gene were targeted by using a pool of 3 shRNA plasmids and then the most potent one was selected (the sense and antisense sequences of STING shRNA were 5′-CCGAATGTTCAATCAGCTATT-3′ and 5′-TAGCTGATTGAACATTCGGTT-3′). Validation of the knock-down was performed at the mRNA level by relative quantitative real-time PCR and at the protein level by western blot. The transfected cells were then treated with PA or control solution at the designated concentrations. Additionally, cells were pretreated with amlexanox at 50 μM for 2 h before PA stimulation.

### Western blot analysis

Lung tissues or cells were homogenized in RIPA buffer containing protease and phosphatase inhibitors. The lysates were centrifuged at 14,000 rpm (15 min, 4 °C) and supernatant was collected for further analysis. Separated with 10% SDS-polyacrylamide gels, the proteins were then transferred to polyvinyl difluoride (PVDF) membrane (Millipore, IPVH00010). Immunoblotting was performed at 4 °C overnight using primary antibodies directed against STING (1:500, Proteintech, 19851-1-AP), TANK-binding kinase 1 (TBK1) (1:1000, Proteintech, 28397-1-AP), phosphorylated TBK1 (phospho-TBK1) (1:1000, Ser172) (Abclonal, AP1026), IRF3 (1:1000, Proteintech, 11312-1-AP), phospho-IRF3(1:1000, Ser396) (Abclonal, AP0623), NF-κB p65 (1:1000, Wanleibio, WL02169), phospho-NF-κB p65(Ser536) (1:1000, Wanleibio, WL01980), β-actin (1:2000, Proteintech, 60008-1-Ig). Membranes were then incubated with secondary antibody (1:10000, SA00001-2, Proteintech) at room temperature for 1 h. Protein bands were detected by ECL solution (E003, 7 Sea Biotech, China) and visualized using the ImageJ software (v.1.53c; NIH).

### Cytokine assay and free fatty acids assay

Lungs were harvested and homogenized using tissue homogenizer. Bronchoalveolar lavage fluid (BALF) was performed by cannulating the trachea with a blunt 22-gauge needle and then lavaging the lungs 3 times with 1 ml of ice-cold PBS. The levels of the TNF-α, IL-6 and interferon β (IFNβ) cytokines in the lung homogenates, BALF supernatant and cell culture supernatant were assayed using commercial ELISA (enzyme linked immunosorbent assay) kits according to the manufacturer’s instructions (Cloud-Clone Corp, China). Lung homogenates were also assayed for free fatty acids using Nonesterified Free Fatty Acids assay kit (Nanjing Jiancheng Bioengineering, China) according to the manufacturer’s protocol.

### Quantitative real-time polymerase chain reaction PCR

Total RNA was isolated from lung tissues using the TRIzol Reagent (Invitrogen). For the quantitative RT-PCR (qPCR), cDNA was synthesized using PrimeScript RT Master Mix (Takara, Japan) according to the manufacturer’s instructions. qPCR was performed on cDNA on ABI 7900HT Real-Time PCR System (Thermo Fisher Scientific Inc.) using TB Green Premix Ex Taq II (Tli RNaseH Plus) (Takara, Japan) and ROX Reference Dye (Takara, Japan). The mRNA levels were calculated by using the comparative threshold method (ΔΔCt) and β-actin as endogenous control. Primers and probes were as follows:

mouse IL-6 forward, 5′-ATGGCAATTCTGATTGTATG-3′;

mouse IL-6 reverse, 5′-GACTCTGGCTTTGTCTTTCT-3′;

mouse TNF-α forward, 5′-CAGGCGGTGCCTATGTCTCA-3′;

mouse TNF-α reverse, 5′-GCTCCTCCACTTGGTGGTTT-3′;

mouse IFNβ forward, 5′-GCTGCGTTCCTGCTGTGCT-3′;

mouse IFNβ reverse, 5′-CGCCCTGTAGGTGAGGTTGA-3′;

mouse β-actin forward, 5′-CTGTGCCCATCTACGAGGGCTAT-3′;

mouse β-actin reverse, 5′-TTTGATGTCACGCACGATTTCC-3′.

### Cytosolic mtDNA quantification

To detect cytosolic mtDNA with qPCR, the cytosol of cultured cells was extracted with a Mitochondria Isolation Kit (Biovision Inc., USA) according to the manufacturer’s instructions. Briefly, after washing with ice-cold PBS, cells treated as indicated were then centrifuged at 600 × *g* for 5 min at 4 °C. The pellet was resuspended in Cytosol Extraction Buffer Mix and incubated for 10 min on ice. The cell suspension was homogenized on ice and centrifuged at 1200 × *g* for 10 min at 4 °C to remove nuclei and unbroken cells. The supernatant was transferred to a fresh 1.5 ml tube and centrifuged at 10,000 × *g* for 30 min at 4 °C. The supernatant was collected as cytosolic fraction. The mtDNA was detected by qPCR with primers that hybridized to sequences in the gene encoding mitochondrial cytochrome c oxidase 1 (mt-Co1). The nuclear DNA was measured with qPCR using primers that hybridized sequences in the 18 S rDNA (coding 18 S ribosomal RNA). The primers for human mt-Co1 were 5′- TTCTTATTTACAGTTGGTGGTC-3′ (forward) and 5′-TCTGAGTAGCGTCGTGGT-3′ (reverse). The primers for 18 S rDNA were 5′-GTAACCCGTTGAACCCCATT-3’ (forward) and 5′-CCATCCAATCGGTAGTAGCG-3′ (reverse). The copy numbers of mtDNA were normalized against the copy numbers of nuclear DNA, and compared between the groups.

### Analysis of mitochondrial membrane potential

To determine the mitochondrial membrane potential following PA treatment, harvested MH-S cells and RAW264.7 macrophages were washed with ice‑cold PBS and incubated with JC-1 staining working solution at 37 °C for 20 min in darkness. Subsequently, cells were washed twice with PBS and analyzed fluorescence intensity of the JC-1 polymer and JC-1 monomer by flow cytometry. The mitochondrial membrane potential was detected as a ratio of fluorescence intensity.

### Statistical analysis

Values were expressed as the mean ± standard deviation (SD). Normality test was determined by Shapiro-Wilk. Two-group comparisons were performed by using two-tailed Student’s *t* test. Multi-group comparisons were performed by using two-tailed ANOVA with post hoc analysis of LSD-t or Games-Howell method. If the data wasn’t fit in normal distribution, two-group comparisons were performed by using the Mann-Whitney test. Differences were considered significant at the two-tailed *P* < 0.05. SPSS version 26 was used for statistical analysis.

## Results

### STING and proinflammatory cytokines in both lungs and BALF were increased in obesity

**Fig. 1:**
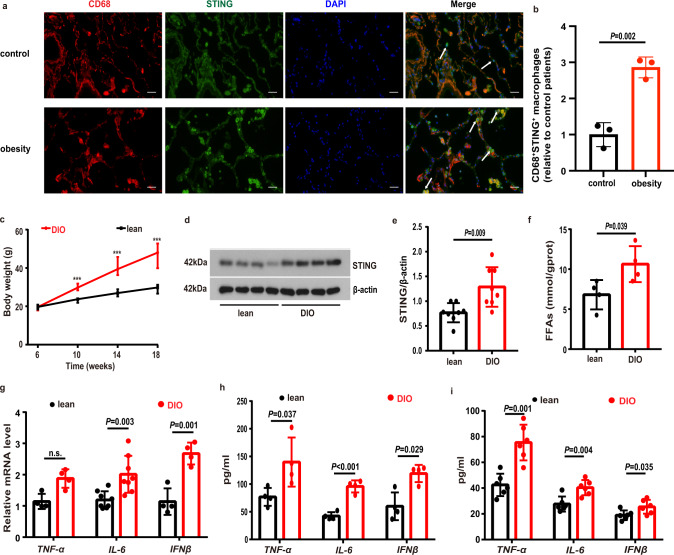
The levels of STING and proinflammatory cytokines were increased in obesity. **a** Representative lung immunofluorescence images of STING^+^/CD68^+^ macrophages from patients with obesity and controls. Scale bars represent 25 μm. **b** Quantification analysis of the numbers of STING^+^/CD68^+^ macrophages in patients with obesity and controls (*n* = 3 each group). **c** Body weight of regular chow (*n* = 14) and high-fat diet (*n* = 15) fed mice. **d**, **e** Representative western blots of STING expressions (**d**), and (**e**) group data of STING fold change in equal amounts of protein extracts of lung tissues from lean (*n* = 8) and DIO (*n* = 9) mice. **f** The levels of free fatty acids (FFAs) in lung tissues were significantly increased in DIO compared to lean mice (*n* = 4 each group). **g** qPCR analysis showed the mRNA levels of TNF-α (*n* = 4), IL-6 (*n* = 8) and IFNβ (*n* = 4) in DIO and control mouse lung tissues. **h** ELISA analysis showed that the protein levels of proinflammatory cytokines in lung homogenates were increased in DIO mice (*n* = 4 each group). **i** ELISA analysis showed that the levels of proinflammatory cytokines in BALF were increased in DIO mice (*n* = 6 each group). Data represent mean ± SD. Statistical significance was determined using two-tailed Student’s *t* test or Mann-Whitney test.

We collected lung tissues from patients with obesity and control individuals. The tissues were sectioned and stained. We observed that the lung sections collected from patients with obesity showed increased numbers of dual STING^+^/CD68^+^ macrophages in lung tissues compared to control individuals (Fig. 1a, b). To define a role of STING in the lung inflammation in obesity, as it currently is not feasible in manipulating signaling pathways in patients, we developed and utilized a high-fat diet (HFD)-induced obese (DIO) mouse model, which were classified following the published criteria including the greater body weight gains in DIO mice compared to controls (Fig. 1c). We observed that the protein expression levels of STING were significantly increased in obese lung tissues compared to controls (Fig. 1d, e). Lung tissues in DIO mice showed increased free fatty acids (FFAs) (Fig. 1f). Meanwhile, the mRNA levels of IL-6 and IFNβ in lungs as well as the protein levels of TNF-α, IL-6 and IFNβ in lung tissue homogenates in obese mice were significantly increased compared to control lean mice (Fig. 1g, h). To confirm the results obtained from the lung tissues, we collected the BALF and observed that the levels of TNF-α, IL-6 and IFNβ in BALF were increased in the BALF from DIO mice compared to control lean mice (Fig. 1i).

### Inhibition of STING alleviated lung inflammation in obesity

**Fig. 2:**
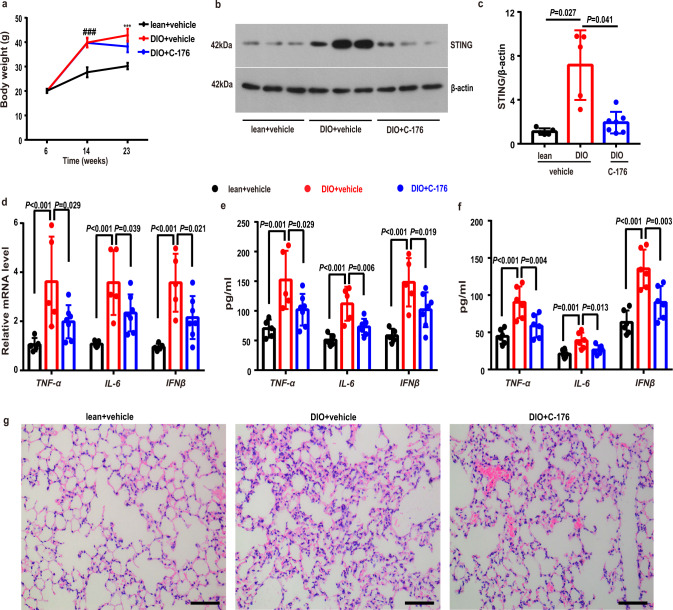
STING inhibition alleviated lung inflammation in obese mice. **a** Body weight of DIO/C-176 (*n* = 13), DIO/vehicle (*n* = 11) and lean/vehicle (*n* = 11) mice groups during regular chow and high fat diet feeding with C-176 or vehicle treatment. **b**, **c** Representative western blots of STING (**b**) and group data of STING (**c**) fold change in equal amounts of protein extracts of mice lung tissues (*n* = 5–7 each group). **d** qPCR analysis showed the mRNA levels of TNF-α, IL-6 and IFNβ in mice lung tissues (*n* = 5–7 each group). **e** ELISA analysis showed that the protein levels of proinflammatory cytokines, including IL-6, TNF-α, and IFNβ, in lung homogenates (*n* = 5–7 each group). **f** ELISA analysis showed the levels of proinflammatory cytokines in mouse BALFs (*n* = 6 each group). **g** Representative H&E images of the lungs in the lean and DIO mice with C-176 or vehicle treatment (*n* = 3 each group). Data represent mean ± SD. ^###^*P* < 0.001, lean + vehicle group vs. DIO + vehicle group. ***p < 0.001, lean + vehicle group vs. DIO + vehicle group, DIO + vehicle group vs. DIO + C-176 group. Statistical significance was determined by one-way ANOVA followed by post-hoc LSD-t or Games-Howell method. Scale bars, 100 μm.

To examine whether inhibition of the STING signaling can prevent obesity-induced inflammation in obesity, we treated mice with a STING inhibitor C-176 or vehicle via intraperitoneal injections once every other day for 9 weeks starting from 14-week HFD feeding. We observed that DIO mice treated with the C-176 showed decreased body weight compared to those treated with vehicle. (Fig. 2a). Consistently, the expressions of STING was remarkably decreased in the C-176-treated mice compared to those vehicle-treated mice (Fig. 2b, c). Matching with the reduced STING expressions, the levels of mRNA and pro-inflammatory cytokines (i.e. IL-6, TNF-α and IFNβ) in lung tissues were predominantly reduced in the C-176-treated DIO mice compared to vehicle groups (Fig. 2d, e). C-176 treatment significantly suppressed the levels of IL-6, TNF-α and IFNβ in BALF (Fig. 2f). Our histological results also showed that inhibition of the STING with C-176 alleviated pathological changes in mouse lung tissues, as evidenced by reduced inflammatory cell infiltration and thickened of alveolar walls (Fig. 2g).

### Metabolic stress disrupted the mitochondrial membrane potential and induced mtDNA leaking into cytosol in MH-S and RAW264.7 macrophages

**Fig. 3:**
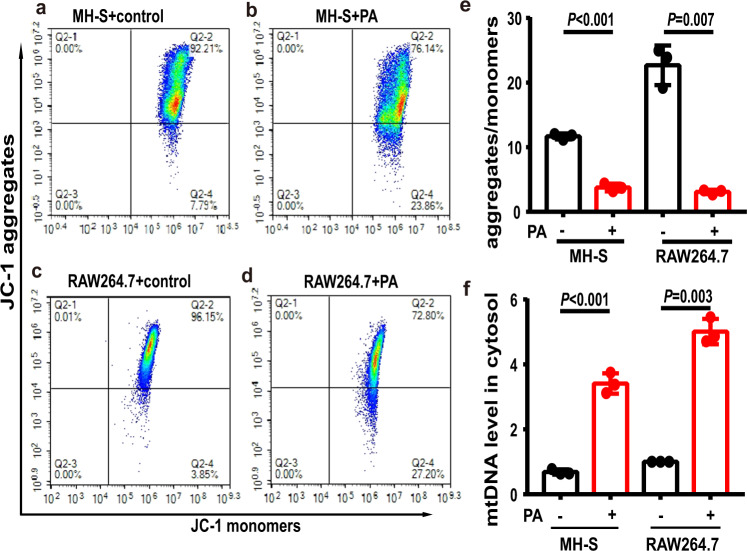
Palmitic acid (PA) significantly reduced mitochondrial membrane potential and increased levels of cytosolic mtDNA in MH-S and RAW264.7 macrophages. **a**–**e** Representative flow cytometry analysis showed the mitochondrial membrane potential of control MH-S cells (**a**), PA-treated MH-S cells (**b**), control RAW264.7 macrophages (**c**) and PA-treated RAW264.7 macrophages (**d**). PA significantly reduced mitochondrial membrane potential (**e**). *n* = 3 biologically independent experiments. **f** PA significantly increased levels of cytosolic mtDNA in MH-S and RAW264.7 macrophages. *n* = 3 biologically independent experiments. Data represent mean ± SD. Statistical significance was determined using two-tailed Student’s *t* test.

There is evidence that the STING is critically involved in inflammation in response to cytosolic mtDNA released from damaged mitochondria [23]. We thus examined mitochondrial functional altervations selectively in the MH-S and RAW264.7 macrophages which were treated with the saturated fatty acid PA. We observed that PA treatment significantly reduced mitochondrial membrane potential in MH-S and RAW264.7 macrophages (Fig. 3a–e). Importantly, we also observed that the amount of mtDNA in the cytosolic fraction was significantly increased in the PA-treated MH-S and RAW264.7 macrophages (Fig. 3f).

### Metabolic stress activated the STING and induced inflammation in MH-S and RAW264.7 macrophages

**Fig. 4:**
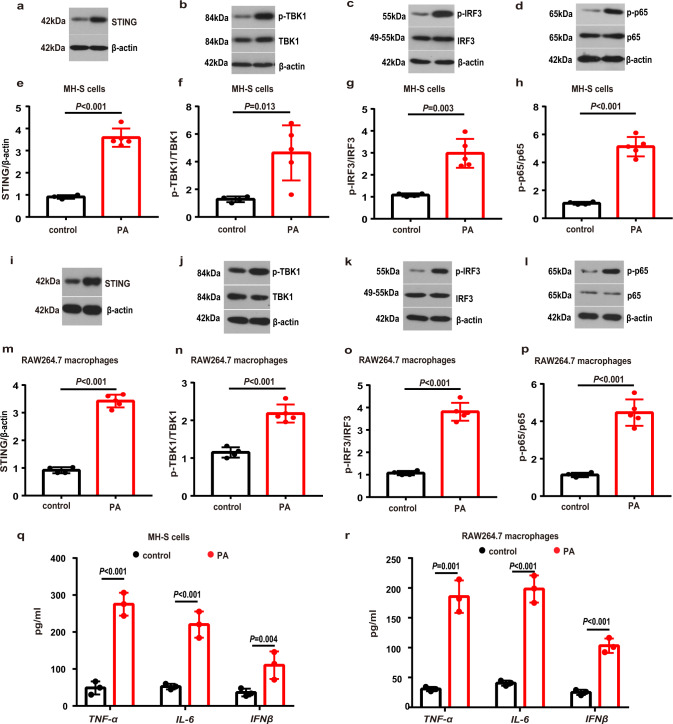
PA activated STING-TBK1-IRF3/NF-κB pathway and induced inflammation in MH-S and RAW264.7 macrophages. MH-S cells were treated with PA (0.6 mM) for 24 h. **a**–**d** Representative western blots of the STING (**a**), p-TBK1 (**b**), TBK1 (**b**), p-IRF3 (**c**), IRF3 (**c**), p-p65 (**d**) and p65 (**d**) in PA-treated MH-S cells. **e**–**h** Group data of STING (**e**), p-TBK1/TBK1 (**f**), p-IRF3/IRF3 (**g**) and p-p65/p65 (**h**) fold change in equal amounts of protein extracts of control (*n* = 4 biological repeats) and PA-treated (*n* = 5 biological repeats) MH-S cells. RAW264.7 macrophages were treated with PA (0.6 mM) for 24 h. **i**–**l** Representative western blots showed the STING (**i**), p-TBK1 (**j**), TBK1 (**j**), p-IRF3 (**k**), IRF3 (**k**), p-p65 (**l**) and p65 (**l**) in PA-treated RAW264.7 macrophages. **m**–**p** Group data of STING (**m**), p-TBK1/TBK1 (**n**), p-IRF3/IRF3 (**o**) and p-p65/p65 (**p**) fold change in equal amounts of protein extracts of control (*n* = 4 biological repeats) and PA-treated (*n* = 5 biological repeats) RAW264.7 macrophages. **q**–**r** ELISA analysis showed that the protein levels of TNF-α, IL-6 and IFNβ in MH-S cells (**q**) and RAW264.7 macrophages (**r**) were significantly increased after PA treatment. *n* = 3 biologically independent experiments. Data represent mean ± SD. Statistical significance was determined using two-tailed Student’s *t* test.

To define a role played by STING signaling in lung macrophage inflammation in PA-induced metabolic stress, we first examined the potential for PA challenge to activate the STING-TBK1-IRF3 /NF-κB pathway. We found that exposure to PA led to a significant increase in the protein expression levels of STING in MH-S cells (Fig. 4a, e) and RAW264.7 macrophages (Fig. 4i, m). Matching with the increased expressions of STING, the phosphorylated TBK1 (p-TBK1) (Fig. 4b, f), the phosphorylated IRF3 (p-IRF3) (Fig. 4c, g) and the phosphorylated NF-κB p65 (p-p65) (Fig. 4d, h) were also augmented in the PA-challenged MH-S cells. These factors were also increased in PA-treated RAW264.7 macrophages (Fig. 4i–p). Consistently, the levels of TNF-α, IL-6 and IFNβ were significnaly elevated in MH-S cells (Fig. 4q) and RAW264.7 macrophages (Fig. 4r) when exposed to the PA.

### The involvement of STING-TBK1-IRF3/NF-κB pathway in the PA-induced inflammation

**Fig. 5:**
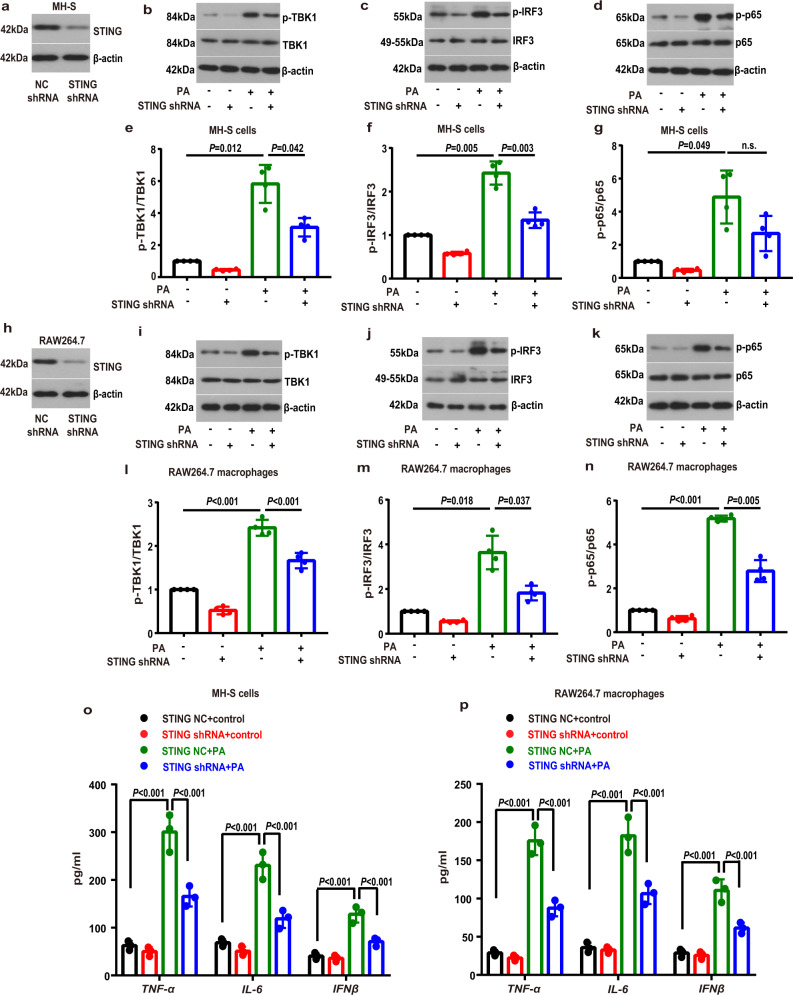
STING knockdown attenuated the PA-induced inflammatory activation in MH-S and RAW264.7 macrophages. The expression of STING was silenced by using specific shRNAs in MH-S (**a**) and RAW264.7 macrophages (**h**). Negative control (NC) shRNA was used in parallel for the control groups. **b**–**g** MH-S cells were transfected with STING shRNA or NC shRNA and then treated with PA (0.6 mM) for 24 h. Representative western blots of the p-TBK1 (**b**), TBK1 (**b**), p-IRF3 (**c**), IRF3 (**c**), p-p65 (**d**) and p-65 (**d**), and group data of p-TBK1/TBK1 (**e**), p-IRF3/IRF3 (**f**) and p-p65/p65 (**g**) fold change in equal amounts of protein extracts of STING shRNA or NC shRNA MH-S cells with or without PA. *n* = 4 biologically independent experiments in each group. **i**–**n** RAW264.7 macrophages were transfected with STING shRNA or NC shRNA and then treated with PA (0.6 mM) for 24 h. Representative western blots of the p-TBK1 (**i**), TBK1 (**i**), p-IRF3 (**j**), IRF3 (**j**), p-p65 (**k**) and p-65 (**k**), and group data of p-TBK1/TBK1 (**l**), p-IRF3/IRF3 (**m**) and p-p65/p65 (**n**) fold change in equal amounts of protein extracts of STING shRNA or NC shRNA RAW264.7 macrophages with or without PA. *n* = 4 biologically independent experiments in each group. **o**, **p** ELISA analysis showed that STING shRNA attenuated the PA-induced increase in levels of proinflammatory cytokines, including TNF-α, IL-6 and IFNβ in PA-treated MH-S cells (**o**) and RAW264.7 macrophages (**p**). *n* = 3 biologically independent experiments. Data represent mean ± SD. n.s. not significant. Statistical significance was determined by one-way ANOVA followed by post-hoc LSD-t or Games-Howell method.

We next defined whether the STING signaling pathway participated in the PA challenge-induced inflammatory activation in lung macrophages. We first deleted the STING in MH-S cells with transfecting MH-S cells with STING shRNA, as evidenced by decreased STING expresssions (Fig. 5a). We then exposed the transduced macrophages to the PA. Expectedly, we observed that genetic knockdown of STING blunted the PA-induced phosphorylation of TBK1 (Fig. 5b, e) and IRF3 (Fig. 5c, f). STING deficiency did not significantly affect phosphorylation of NF-κB p65 in PA-treated MH-S cells (Fig. 5d, g). Similarly, we transduced RAW264.7 macrophages with the STING shRNA (Fig. 5h), and also observed that knockdown of STING blunted the PA-induced effects on TBK1 (Fig. 5i, l), IRF3 (Fig. 5j, m) and NF-κB p65 (Fig. 5k, n). Furthermore, downstream proinflammatory cytokines, including TNF-α, IL-6 and IFNβ, were notably lower in the STING-deleted MH-S cells (Fig. 5o) and RAW264.7 macrophages (Fig. 5p) than controls.

**Fig. 6:**
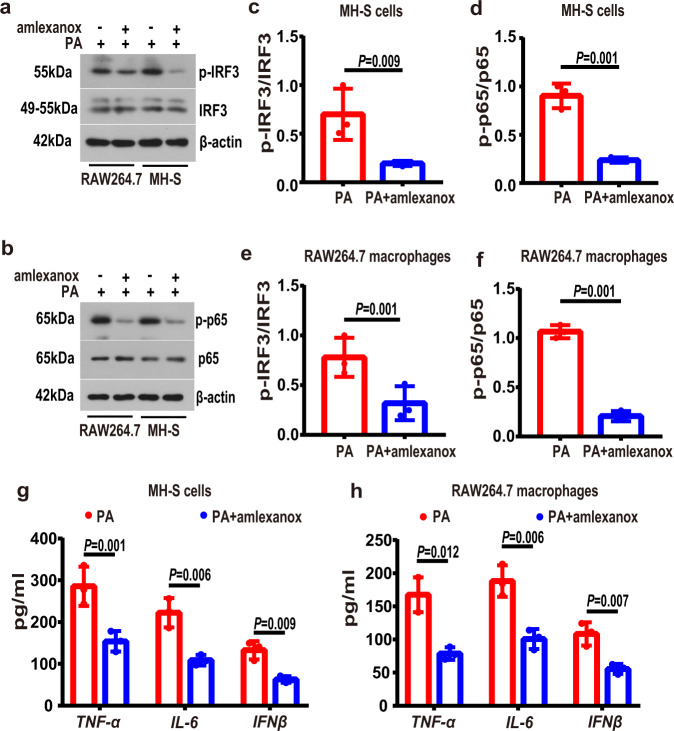
TBK1 inhibition alleviated inflammatory activation in PA-treatment MH-S and RAW264.7 macrophages. MH-S and RAW264.7 macrophages were treated with PA (0.6 mM) for 24 h in the absence or presence of amlexanox (50 μM, 2 h before PA stimulation). **a**, **b** Representative western blots of the p-IRF3 (**a**), IRF3 (**a**), p-p65 (**b**) and p-65 (**b**) in PA-treated MH-S cells and RAW264.7 macrophages with or without amlexanox. **c**–**f** Group data of p-IRF3/IRF3 (**c**, **e**) and p-p65/p65 (**d**, **f**) fold change in equal amounts of protein extracts of PA-treated MH-S cells and RAW264.7 macrophages with or without amlexanox. *n* = 3 biologically independent experiments in each group. **g**, **h** ELISA analysis showed that amlexanox decreased the levels of proinflammatory cytokines, including TNF-α, IL-6 and IFNβ in PA-treated MH-S cells (**g**) and RAW264.7 macrophages (**h**). *n* = 3 biologically independent experiments. Data represent mean ± SD. Statistical significance was determined using two-tailed Student’s *t* test.

In parallel, we defined the role of TBK1, a downstream factor of STING signaling pathway, in obese lung macrophagic inflammation, with a TBK1 inhibitor amlexanox [24]. Consistently, we observed that amlexanox treatment reduced the PA-induced increase in the phosphorylation levels of of IRF3 and NF-κB p65 (Fig. 6a–f), and reduced the levels of proinflammatory cytokines (Fig. 6g, h) in the PA-treated MH-S and RAW264.7 macrophages.

## Discussion

There is ample evidence to show that obesity is associated with a low-grade proinflammatory state [12, 13], referred to as “metaflammation” [11, 25, 26]. The obesity-related molecular mediators could significantly alter inflammatory responses in the lung, leading to the susceptibility to some lung diseases [9, 14, 27]. In this study, we defined a previously unknown role of the STING in obesity-induced lung inflammation. Our data demonstrate that the STING and its downstream signaling pathways in macophages are invovled in the inflammatory processes in obese lungs. The STING is known to be involved in regulating the immune response of various pulmonary inflammatory diseases [17–20]. Recent studies show that the STING signaling pathway provides a link between metabolism and immunity [28]. Meanwhile, emerging evidence suggests that the STING signaling in macrophages plays a vital role in the obesity-induced inflammation and metabolic disorders [21, 29–32].

Our clinical results demonstrate that patients with obesity have increased levels of STING in human CD68^+^ lung macrophages compared to control individuals. Consistently, the protein levels of STING and proinflammatory cytokines such as TNF-α, IL-6 and IFNβ were increased significantly in lung tissues and BALF in obese mice. The STING is known to initiate effective type I IFNs production including the IFNβ [33, 34]. Recent studies show that obesity-driven induction of the type I IFNs contributes to obesity-associated pathogenesis in mice [35, 36]. Our data also showed that inhibition of the STING remarkably reduced the level of inflammatory cytokines and alleviated pathological alterations in lung tissues in obese mice. We thus posited that STING signaling in lung macrophages partakes in the obesity-induced lung inflammation.

Palmitic acid (PA), the most representative FFA in obesity, is well accepted to mimic in vitro metabolic stress conditions of DIO [37–39]. As it is challenging to manipulate such signaling pathways in patients, we explored the underlying mechanism of STING in obese lung from PA-treated MH-S cells and RAW264.7 lung macrophages in vitro. We found that PA challenge promoted the release of mtDNA into the cytoplasm in lung MH-S cells and RAW264.7 macrophages. Mitochondrial dysfunctions are associated with metabolic syndrome [40–43]. Our data show that PA-based metabolic stress potently decreased mitochondrial membrane potential, a potential reason of mtDNA release. Increased mtDNA in other tissues and plasma in obesity or metabolic stress could induce proinflammation [44, 45] and activate the STING signaling pathway, an aberrant DNA sensor in cytoplasm [21, 46].

Metabolic stress induced release of the mtDNA leakage in lung macrophages would probably stimulate the STING signaling pathways. It has been well documented that STING stimulates the expressions of genes involved in host immune response by promoting the phosphorylation and translocation of several transcription factors, including NF-κB, IRF3 and signal transducer and activator of transcription 6 (STAT6) [15, 16, 47]. In the current study, we provided compelling evidence to show that the STING played a deleterious role in metabolic stress-induced proinflammatory activation, and observed that STING deletion ameliorated PA-induced inflammation, accompanied by inactivation of TBK1-IRF3/NF-κB signalling in RAW264.7 macrophages and inactivation of TBK1-IRF3 signalling in MH-S cells. The deletion of STING in PA-treated MH-S cells did not significantly inhibit phosphorylation of NF-κB p65, which might be due to the partial activation of NF-κB sigalling by STING in MH-S cells. In addition, it is well known that the two signaling arms, IRF3/NF-κB pathways, are thought to be mediated by a single upstream kinase TBK1 [48]. Our data also show that inhibition of TBK1 led to a significantly decrease in phosphorylation of IRF3 and NF-κB p65 and also had a protective role in PA-induced inflammation in MH-S and RAW264.7 macrophages. We assumed that the STING is involved in activating metabolic inflammation via TBK1-IRF3/NF-κB pathway in lung macrophages.

We are aware that there are some limitations in the current study. For example, different types of macrophages in lung tissues play complex roles in the mechanism, while our findings do not allow us to definitively determine the different roles of STING in pulmonary interstitial macrophages and alveolar macrophages of obesity. Also, the number of the participants were limited. The potential biomarkers of respiratory tract related to STING should also be further explored in the future.

Collectively, our data reveal that activated STING in obesity enhances lung macrophages proinflammatory ability, a potential mechanism underlying obesity-ralated lung inflammation. The STING signaling pathway would probably be pharmacologically targetable to ameliorate obesity-related lung diseases.

## Supplementary information

Supplementary Table S1

## Data Availability

All the data generated and analyzed that support the findings in this study are within the article and its supplementary information files, and are available from the authors upon reasonable request.
