# Supplementary material for: A role of STING signaling in obesity-induced lung inflammation
Source: Int J Obes (Lond). 2023 Feb 13;47(4):325–34. doi: 10.1038/s41366-023-01272-x (PMC9924210; doi:10.1038/s41366-023-01272-x)
Supplement: Supplementary file 1 — Supplementary Table S1 [file 41366_2023_1272_MOESM1_ESM.docx]

Supplementary Table S1. The basic clinical characteristics of recruited patients.

|  | Control group | Obesity group | *P*-value |
| --- | --- | --- | --- |
| Diagnosis | adenocarcinoma (n=2)  inflammatory pseudotumor (n=1) | adenocarcinoma (n=2)  granuloma (n=1) |  |
| Age (years) | 55.0±12.8 | 51.7±7.6 | 0.718 |
| Sex | 2 males  1 female | 1 male  2 females | 0.414 |
| BMI (kg/m^2^) | 21.56±2.03 | 30.58±0.72 | 0.002** |

**Supplementary Table S1. The basic clinical characteristics of recruited patients.**

Baseline characteristics of control group and obesity group. BMI, body mass index; Data were expressed as mean ± SD, **: *P* < 0.01.
